# Comparison of methods to estimate water access: a pilot study of a GPS-based approach in low resource settings

**DOI:** 10.1186/s12942-016-0062-8

**Published:** 2016-09-20

**Authors:** Amber L. Pearson

**Affiliations:** 1 Department of Geography, Environment and Spatial Sciences, Michigan State University, East Lansing, MI 48824 USA; 2 Environmental Science and Policy Program, Michigan State University, East Lansing, MI 48824 USA; 3 Department of Public Health, University of Otago, Wellington, 6242 New Zealand

**Keywords:** GPS, Access, Water, Self-report, Euclidean distance, Africa, Methodology

## Abstract

**Background:**

Most water access studies involve self-reported measures such as time spent or simple spatial measures such as Euclidean distance from home to source. GPS-based measures of access are often considered *actual* access and have shown little correlation with self-reported measures. One main obstacle to widespread use of GPS-based measurement of access to water has been technological limitations (e.g., battery life). As such, GPS-based measures have been limited by time and in sample size.

**Methods:**

The aim of this pilot study was to develop and test a novel GPS unit, (≤4-week battery life, waterproof) to measure access to water. The GPS-based method was pilot-tested to estimate number of trips per day, time spent and distance traveled to source for all water collected over a 3-day period in five households in south-western Uganda. This method was then compared to self-reported measures and commonly used spatial measures of access for the same households.

**Results:**

Time spent collecting water was significantly overestimated using a self-reported measure, compared to GPS-based (p < 0.05). In contrast, both the GIS Euclidean distances to nearest and actual primary source significantly underestimated distances traveled, compared to the GPS-based measurement of actual travel paths to water source (p < 0.05). Households did not consistently collect water from the source nearest their home. Comparisons between the GPS-based measure and self-reported meters traveled were not made, as respondents did not feel that they could accurately estimate distance. However, there was complete agreement between self-reported primary source and GPS-based.

**Conclusions:**

Reliance on cross-sectional self-reported or simple GIS measures leads to misclassification in water access measurement. This new method offers reductions in such errors and may aid in understanding dynamic measures of access to water for health studies.

## Background

Adequate and safe access to water is considered essential for health and was responsible for an estimated 17.4 % of deaths in children 1–11 months globally in 2010 [1]. High rates of child and infant deaths means that life expectancy in sub-Saharan Africa is startlingly low at 58 years [2]. Yet, surprisingly, we still lack a realistic understanding of day-to-day access to water, as a dynamic process across days and seasons.

While headway has been made to improve access to water, research has shown that global estimates of population access to water are overestimated due to the way access has been measured to date [3]. Estimated global expenditure on water and sanitation is an enormous $28.4 billion per year [4]. Yet, in sub-Saharan Africa, an estimated 1 in 3 well hand pumps are non-functional [5]. Still, almost all water-health research measures self-reported access to water at one point in time or distance to nearest water source. In reality, access to water is a dynamic process, often shifting with seasons [3], water availability [6], in response to failing infrastructure [7, 8] or eco-social processes which may influence access [9]. Such inaccessibility can lead to changes in water source type with implications for health. Likely, in reality, far less of the population has easy access to safe and reliable water than global estimates suggest. In order to improve access to water globally, we must first understand its dynamic nature. A primary obstacle to measuring and understanding the dynamic nature of access has been limitations of spatial technologies.

To date, there have been several major obstacles to accurate and extensive GPS data on spatial dimensions of access. Largely, the limitations have related to inadequate battery life, cumbersome data retrieval, weighty/bulky GPS units, and significant noise in the spatial data. For example, most studies have used GPS technology that requires daily contact with the respondent or requires the respondent to power on/off the unit [10]—both of which can result in data loss, bias, or high research costs. Some researchers report testing different units [10] and identified that of all low-cost GPS units, a battery life of 20 h with data retrieval using a USB connection was the best available on the market in 2011 (called Holux M-1000C, ~$70), although not waterproof. Given these technological limitations, collection of precise, spatial measures on a large number of respondents over time has proven elusive.

GPS technologies have been used to measure access in a few contexts, although measurements have been prone to bias and error. At times, spatial dimensions of access are highly correlated with self-reported measures and at times not at all [10, 11]. In their simplest form, spatial access measures may be the Euclidean distance from a respondent’s home to a water source (most often the nearest source) identified through GPS or aerial/satellite imagery. Such measurement is problematic because: (a) the respondent may not have access to/use the nearest source; and (b) the measure does not account for route traveled or topography, impacting both distance and time to fetch water. In fact, little correlation between self-reported time to fetch water and GPS-generated distance measures have been observed [11]. Likewise, some research indicates low correlation between self-reported minutes to collect water and GPS-recorded time to collect water, whereby self-reported minutes were significantly higher [10]. A plausible and important reason for the lack of correlation between self-reported measures and spatial measures may relate to limitations in spatial data collection methods employed. Although very informative, the few previous methodological studies of spatial access to water lacked: (i) comparison beyond Euclidean distance and assumed path (not GPS-based) [11]; (ii) comparisons between self-reported and spatial measures within the same households [10]; (iii) comparison of Euclidean and GPS measures for more than a one day or single water collection trip [12]; and (iv) comparison of all three (self-reported, GPS-based and Euclidean) access measures [10–13].

The novelty of the current study is twofold. First, this study improves upon previous studies as it: (i) directly compares self-reported and GPS-based and Euclidean distance measures in the *same* households; (ii) involves GPS measurement of actual travel path from home to source rather than the path selected by a local leader or via satellite imagery; and most importantly (iii) captures water collection practices over several days, rather than a single trip or to only one source. Second, the GPS technology developed for this study is novel in terms of its extended battery life (up to 4 weeks), small size, relatively inexpensive components (~$250), waterproof plastic casing and non-impedance of the jerry can handle—which allow for longer observation periods.

This method was employed to measure aspects of access to water over a 3-day period in south-western Uganda and then compared with self-reported and Euclidean GIS measures. Specifically, this study compared: (1) minutes spent per roundtrip; (2) distance traveled per one-way trip; and (3) whether self-reported primary source type corresponded with GPS-based source most frequently visited over the study period.

## Methods

### Study site

The study village was located in the semi-arid savannah of rural, south-western Uganda in Kiruhura District. The region has an annual extended dry season (June–September), yet high rainfall during the wet season (mean = 1000 mm) [14]. In addition to this physical environment, the lack of government provision of improved water sources, the lack of maintenance of water sources, and the dispersed nature of the communities means that water access is generally poor.

Using household data (n = 62) from a previous study in Rwamuhuku village [8], Euclidean distance from household location to primary water source used in the wet season was used to purposely select five households with varying previously calculated distances to water source (median = 652 m, mean = 949 m, SD = 818 m). In theory, households near sources may visit them more frequently, and households far from sources may visit the sources less frequently. Thus, a total of five households was selected from the 90th percentile in the near and far groups. This study was conducted in May 2016, near the end of the rainy season.

### GPS unit specifications and deployment

**Fig. 1:**
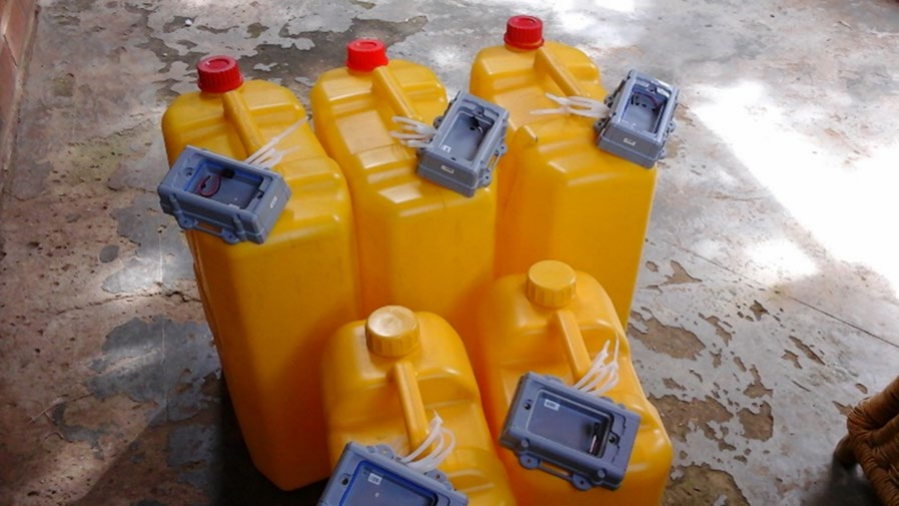
GPS-enabled jerry cans (20 and 10 L) with batteries awaiting installation

The GPS unit electronic components were developed by Sparx Engineering (Houston, TX). The device was designed to rest in sleep mode and to ‘wake up’ to collect and store GPS coordinates when the device is in motion. It has timeouts for sensing motion so that the device can stay in a low power state for as long as possible. When the device senses motion for long enough, it powers on the GPS and starts saving data to the internal microSD card. There are no buttons or lights on the exterior of the unit, so there is no indication visual or otherwise that the device is operational. This is to reduce potential user interest or tampering. Once the logger stops moving for 20 s, the GPS sensor goes into sleep mode and thus into a low power state. Bespoke software was developed to log the GPS readings to the microSD card in an easily parsed comma separated text file. Including the water-proof plastic casing, the unit weighed a total of 187 g. These units were attached to 10 and 20 L jerry cans using cable ties, explicitly allowing for use of the jerry can handle (Fig. 1).

After enrollment, the household head was asked which household member was the primary person to collect water for the household. Then, the appropriate size of jerry can was selected (20 vs. 10 L), since young children are unable to carry the large jerry cans. Each household was then given a GPS-enabled jerry can and members were instructed on its use. Instructions included: (a) Please collect water as usual over the next 3 days; (b) Please use this jerry can to collect water; (c) Store jerry can inside home, as usual, when not in use; (d) Please do not play with the jerry can or disturb it unless collecting water; and (e) Please do not use the jerry can for other purposes. GPS data were then collected for 3 days (mid-Tuesday to mid-Friday).

### Survey of water collection practices and self-reported access measures

A survey was then conducted in each home to capture self-reported dimensions of access including: (a) primary water source; (b) roundtrip time spent per trip; and c) one-way distance to primary source. GPS coordinates were collected for home locations and all water sources. These data were then used to calculate Euclidean distance to nearest source and to primary source, in ArcGIS v10.2 (Redlands, CA, USA), as these are commonly used measures of access to water. In addition, other aspects of water collection practices which may influence the ability to scale-up this pilot study were obtained including activities conducted while collecting water, socializing, laundry practices, and businesses run from the home.

### Analytical comparison of self-reported or simple GIS measures and GPS-based measures

To compare measures, a variety of non-parametric statistical tests were used, due to the small size of the pilot sample. To compare minutes spent per roundtrip between self-reported and GPS-based measures, the Wilcoxon signed rank test was used. To compare differences between GPS-based distances and either GIS Euclidean to primary source or GIS Euclidean to nearest source, the Wilcoxon signed rank test was used. Last, to compare differences in counts of households reporting the use of more than one source in a day, compared to GPS-based measures, Chi square fisher’s exact test was used. All analyses were conducted in Stata v13 (College Station, TX, USA).

## Results

First the characteristics of water collectors and collection practices were examined. In the small pilot sample (n = 5 households), 60 % of the water collectors were female and 60 % were adults (Table 1). Only one respondent reported completing other activities while collecting water—playing with friends. All but one respondent reported waiting in a queue at the water source (80 %). Most respondents reported collecting water with friends and family, and these were the youngest respondents. The two children reported carrying the 10 L jerry can, whereas the three adults reported carrying the 20 L can. All but the eldest respondent reported carrying more than one jerry can per collection trip. The two respondents that reported collecting water from multiple sources also reported using that water for different purposes. All respondents reported washing laundry on Saturday. Four of the five respondents also washed laundry on other days including Monday, Tuesday and Wednesday.

**Table 1:** Descriptive statistics on water collection practices

Characteristics	N	%
Water collector—child (< 18 years)	2	40
Water collector—adult (18 + years)	3	60
Water collector—male	2	40
Water collector—female	3	60
Performs other activities during water collection	1	20
Typically waits in queue	4	80
Collects water with friends/family	3	60
Maximum size jerry can carried, 10 L	2	40
Maximum size jerry can carried, 20 L	3	60
Typically collects more than one jerry can	4	80
Water collected from different sources used for different purposes	2	40
Laundry typically done on—Saturday (and possibly other days)	5	100
Laundry typically done on two days per week	4	80

Over the observation period, the roundtrip water collection trips ranged from 2–8 times, with a daily average for the sample of 1.4 trips (Table 2). On average, queue and water filling times were 6.8 min (SD = 6.3).

**Table 2:** Descriptive statistics of total trips and queue/filling time

Number of GPS-based return trips over observation period by household	N
Household 1	8
Household 2	4
Household 3	4
Household 4	2
Household 5	3
Number daily return trips, mean	1.4
Queue min per trip, mean (SD)	6.8 (6.3)

**Fig. 2:**
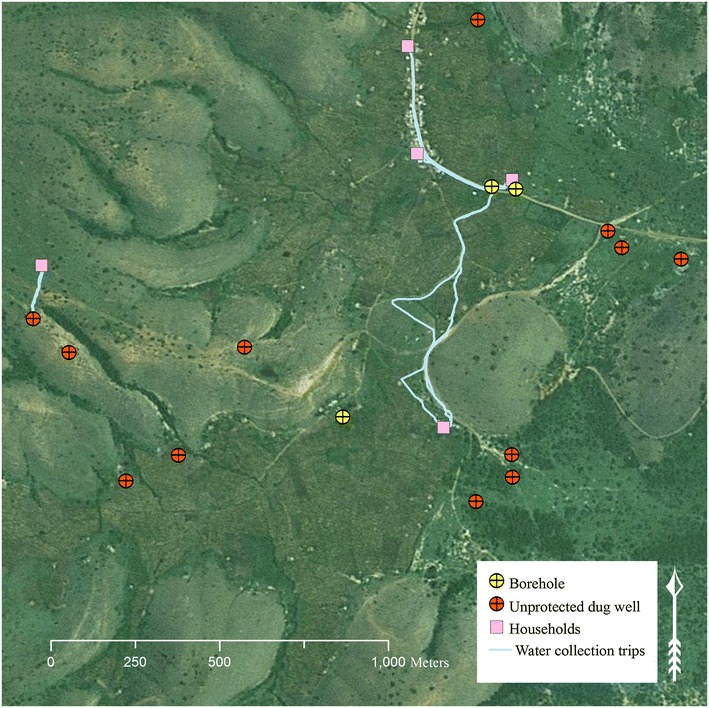
Map of water collection trips, water source locations and homes

When comparing distance traveled and time spent collecting water, there was clear differences between GPS-based measures and both self-reported and simple GIS measures (Table 3). Self-reported minutes traveled was significantly higher than the GPS-based measure of minutes traveled (p < 0.05). In contrast, both the GIS Euclidean distances to nearest and actual primary source were significantly lower than the GPS-based measurement of actual travel paths to water source (p < 0.05), indicating that households do not always use the source nearest their home. This is visible in a map of water collection activities, locations of water sources and homes (see Fig. 2). Comparisons between the GPS-based measure and self-reported meters traveled were not made, as respondents did not feel that they could accurately estimate distance.

**Table 3:** Comparison of measures—time spent and distance traveled

	Measure 1	GPS-based measure
Minutes spent per roundtrip including queue/fill time, mean (SD)^a^	66 (39.1)	18.5 (24.7)
Meters traveled per one-way trip, mean (SD)^b^	335.1 (267.5)	397.8 (345.5)
Meters traveled per one-way trip, mean (SD)^c^	174.4 (86.5)	397.8 (345.5)

All differences were significant at the p = 0.05 level

^a^Measure 1 = Self-reported; Wilcoxon signed rank test used

^b^Measure 1 = GIS Euclidean distance to primary source; Wilcoxon signed rank test used

^c^Measure = GIS Euclidean distance to nearest source; Wilcoxon signed rank test used

Lastly, there was complete agreement between self-reported primary source type and GPS-based most frequently visited source, whereby four households primarily used and reported using a borehole and one household used an unprotected hand dug well (Table 4).

**Table 4:** Comparison of measures—primary water source used

	Self-report		
	Borehole	Unprotected spring	Unprotected dug well
*GPS-based*			
Borehole	4	0	0
Unprotected spring	0	0	1
Unprotected dug well	0	0	0

## Discussion

The aim of this research was to develop and test a novel GPS technology and to compare measurements of access to water between the GPS-based measures and other commonly used measures including self-report and simple GIS measures. Self-reported time to collect water was significantly higher than our GPS-based measurement. Simple GIS measures of distance to source was significantly lower than our GPS-based measures. The implications for these findings are that water access is likely significantly over- and under-estimated depending on which commonly used measure is employed. Our novel GPS-based measurement offers not only the potential to reduce these measurement errors, but also to do so over a longer period of time (due to extended battery life) and in a large sample (due to the low cost and external simplicity of the design). Other studies that have employed GPS measurement of water access have done so in small samples and for a short period of time. Based on the results of this pilot study, it appears that this new methodology may be ready to scale-up and be used in larger longitudinal studies of the dynamics of access to water. However, careful considerations must be made based on context.

In this particular community, both males and females and adults and children collect water. Therefore, the size of the container used to collect water may vary and this must be considered during deployment of the units. In addition, one respondent reported conducting other activities en route to collect water, which may bias GPS-based measures of access. This requires careful consideration prior to deployment. It may be an undue burden on participants to request that they only collect water during a trip. Therefore, a weighting scheme on percentage of time or distance explicitly spent collecting water may be needed. Likewise, water collection may take longer than ‘necessary’ if done so with friends or family members. In other words, socializing while collecting water may mean slower walking times. However, these measurements of time are perhaps more realistic. Last, one dimension of water access not assessed here is water quantity obtained/used. Because households typically use more than one container to collect water and because GPS units were only fitted to one container per household, reliable estimates of water obtained could not be calculated. If future studies are particularly interested in water quantity collected as an indicator of access, consideration must be made about the timing of data collection (e.g., days on which laundry is washed), the number and size of containers used to collect water, and whether or not households operate a business/garden from home which may require water.

Misclassification in water access measurement will lead to inaccurate conclusions about the water-health relationship. This study indicates that if self-reported time were used to measure access, the effect size of water access on health may be over-estimated. In contrast, this study indicates that if Euclidean distances were used, the effect size would be under-estimated. As such, using the GPS-based method reduces this bias and serves as an objective yet realistic method for measuring water access.

### International significance and impact

There are important international implications of our finding that households did not use the source nearest their home or used sources other than a borehole (surface water) even when a borehole was present. This finding highlights the likely over-estimation of populations with access to improved water, most probably in many semi-arid, low resource settings. In fact, most of the estimated 1–2 billion people currently facing water insecurity reside in the drylands [15]. Drylands and other regions with climate-driven changes in rainfall patterns may increasingly be experiencing similar inaccessibility of ‘available’ water sources, as found in this study. Because the Joint Monitoring Program defines access to improved water as the presence of “public boreholes, protected dug wells, protected springs, rainwater collection” [16], but does not measure whether water is available or whether the water infrastructure is functioning, global estimates of access are likely inflated. The consequences of over-estimating access to water may include the reduction in funding allotted for water provision in areas which actually need infrastructure improvements. This novel GPS technology may contribute to more realistic global estimates.

The GPS technology developed in this study is internationally significant as it offers a relatively low cost, precise measurement of dynamic water access. The long battery life allows the technology to be deployed for longer study periods, with no reliance on the user. This means that similar studies could be carried out in other low resource settings, in a larger sample, and over a longer period than previous studies [10–13].

The spatial data gap (for water and beyond) in low resource settings has been highlighted as an obstacle to informed decision-making, and was recently designated as an investment sector of the Bill & Melinda Gates Foundation in August 2016 [17]. The GPS-based method tested here could contribute to filling our global knowledge gap in the spatial dimensions of water access. In addition, such data could uncover actual water infrastructure failures, currently estimated to be 20–40 % in Africa, South Asia and Central America [18]. While the GPS-based method in this study was employed in Uganda, it should be tested in many other low- and middle-income countries and in larger samples over time to confirm these findings. Such findings could improve global population estimates of those with easy access to reliable water, and thus inform international decision-making, as we embark upon efforts to meet the post-2015 Sustainable Development Goals.

## Conclusion

Reliance on cross-sectional self-reported measures or simple GIS measures leads to misclassification in water access measurement. This new GPS-based method offers reductions in such errors as it allows for measurement of actual water collection behaviors over time. Such findings may aid in understanding dynamic measures of access to water for health studies.

## Abbreviations

GIS: Geographic Information System
GPS: Geographic Positioning System
USB: Universal Serial Bus
